# Systematic review on factors influencing the effectiveness of alcohol-based hand rubbing in healthcare

**DOI:** 10.1186/s13756-021-01049-9

**Published:** 2022-01-24

**Authors:** Lesley Price, Lucyna Gozdzielewska, Julius Cesar Alejandre, Annelysse Jorgenson, Emma Stewart, Didier Pittet, Jacqui Reilly

**Affiliations:** 1 SHIP Research Group, Research Centre for Health, Glasgow Caledonian University, Cowcaddens Road, Glasgow, G4 0BA Scotland, UK; 2 School of Computing, Engineering, and Built Environment, Glasgow Caledonian University, Cowcaddens Road, Glasgow, G4 0BA Scotland, UK; 3 MRC/CSO Social and Public Health Sciences Unit, University of Glasgow, Berkeley Square, 99 Berkeley Street, Glasgow, G3 7HR UK; 4 Infection Control Programme and WHO Collaborating Center on Patient Safety, The University of Geneva Hospitals and Faculty of Medicine, Geneva, Rue Gabrielle Perret-Gentil, 1211, Geneva 4, Switzerland

**Keywords:** Hand hygiene, Alcohol-based handrub, Systematic review, Volume, Application time

## Abstract

**Background:**

The effectiveness of hand rubbing with alcohol-based handrub (ABHR) is impacted by several factors. To investigate these, World Health Organization (WHO) commissioned a systematic review.

**Aim:**

To evaluate the impact of ABHR volume, application time, rubbing friction and hand size on microbiological load reduction, hand surface coverage or drying time.

**Methods:**

Medline, CINAHL, Web of Science and ScienceDirect databases were searched for healthcare or laboratory-based primary studies, published in English, (1980- February 2021), investigating the impact of ABHR volume, application time, rubbing friction or hand size on bacterial load reduction, hand coverage or drying time. Two reviewers independently performed data extraction and quality assessment. The results are presented narratively.

**Findings:**

Twenty studies were included in the review. Categories included: ABHR volume, application time and rubbing friction. Sub-categories: bacterial load reduction, hand size, drying time or hand surface coverage. All used experimental or quasi-experimental designs. Findings showed as ABHR volume increased, bacterial load reduced, and drying times increased. Furthermore, one study showed that the application of sprayed ABHR without hand rubbing resulted in significantly lower bacterial load reduction than poured or sprayed ABHR with hand rubbing (− 0.70; 95%CI: − 1.13 to − 0.28). Evidence was heterogeneous in application time, volume, technique, and product. All studies were assessed as high risk of bias.

**Conclusions:**

There is insufficient evidence to change WHO recommendation of a palmful of ABHR in a cupped hand applied for 20–30 s or manufacturer-recommended volume applied for about 20 s (Centers for Disease Control and Prevention). Future hand hygiene research should standardise volume, application time, and consider hand size.

**Supplementary Information:**

The online version contains supplementary material available at 10.1186/s13756-021-01049-9.

## Background

The World Health Organization (WHO) identifies effective hand hygiene as one of the most important methods for preventing infection transmission [1, 2]. The effectiveness of hand hygiene may be impacted by the hand hygiene product and application technique at the recommended times during clinical practice [3].

Technique for the application of ABHR, product and moments for hand hygiene are well-described parameters in extant guidance for hand hygiene [2, 4]. However, with regards to the volume of ABHR, hand hygiene guidelines do not provide definitive recommendations. WHO [2] recommends that a “palmful of the product sufficient to cover all hand surfaces” should be used, while Centers for Disease Control and Prevention (CDC) [4] indicates that the manufacturer-recommended volume should be applied. With respect to the duration of hand rubbing, WHO [2] recommends 20–30 s application time, while CDC [5] rubbing hands until dry for around 20 s. However, the difference between 20 and 30 s could have an impact on the effectiveness of hand rubbing. Furthermore, it has been suggested that both ABHR volume and duration of hand rubbing appear to be linked to hand size [3]. Larger hands have a bigger surface area that needs to be covered with ABHR; thus, might require a greater amount of ABHR to achieve complete surface coverage and to keep both hands wet for the recommended 20–30 s. Rubbing friction has also been indicated as an important consideration for the efficacy of ABHR application in reducing the microbiological load on hands, possibly because rubbing friction helps to dislodge the bacteria from the surface of the hands resulting in an increased exposure of the microorganisms to the ABHR [6].

Systematic reviews exist on the interventions for improving compliance with hand hygiene [7], and on the effectiveness of hand hygiene technique [8], but none to the authors’ knowledge of other factors influencing hand hygiene effectiveness. There is a need to better understand the impact of these recognised factors on hand hygiene effectiveness, to identify the optimal duration of hand rubbing, and to determine the volume of ABHR that should be used to prevent infection transmission in healthcare. This systematic review aimed to evaluate the impact of ABHR volume, application time, rubbing friction and hand size on microbiological load reduction, hand surface coverage or ABHR drying time.

## Methods

This systematic review was registered with the international prospective register of systematic reviews (PROSPERO 2021: CRD42021236142) (Available from: https://www.crd.york.ac.uk/prospero/display_record.php?ID=CRD42021236142). The review is reported in accordance with the Preferred Reporting Items for Systematic Reviews and Meta-Analyses (PRISMA) statement [9].

### Inclusion criteria

The review considered studies with human participants in the context of healthcare practice or laboratory settings and focusing on hand rubbing with ABHR in relation to any of the following factors: ABHR volume, ABHR application time, rubbing friction exerted during hand rubbing or hand size. ABHR was operationalised as an alcohol solution, either in a liquid, gel or foam format, designed for application to the hands to reduce bacterial load on hands, as per WHO definition [2]. Outcomes of interest were bacterial load reduction on hands, hand surface coverage with ABHR or ABHR drying time.

### Exclusion criteria

Studies conducted in healthcare settings in which participants were patients or visitors and research conducted within operating theatres focusing on surgical hand antisepsis were excluded. Furthermore, studies focusing on handwashing with soap and water or investigating the effect of wearing long, varnished or artificial nails or hand jewellery, the use of gloves or with outcomes related to compliance with hand hygiene opportunity or technique or skin tolerance were not deemed eligible because these factors are not directly associated with the process of ABHR application. Finally, studies evaluating the effectiveness of ABHR products or hand rubbing technique were not considered for inclusion because systematic reviews on the effectiveness of these factors have already been conducted [8, 10].

### Types of study

The review considered all empirical research designs, including randomised controlled trials (RCTs), non-randomised trials (NRTs), before and after studies, case–control studies, cohort studies and observational descriptive studies. Reviews and non-primary research records, such as editorials, opinion-based papers and commentaries were excluded.

### Search strategy

A three-stage search strategy was employed. Search terms related to ABHR, volume, time, rubbing friction, hand size, bacterial load and hand surface coverage were searched in MEDLINE, CINAHL, ScienceDirect and relevant databases on the Web of Science gateway; namely the Web of Science Core Collection, Scientific Electronic Library Online (SciELO) Citation Index, and Korean Journal Database (KCI). The search was restricted to sources published in the English language and the review covers the period between 1980 and February 2021. The limit of sources published since 1980 was applied because hand rubbing with ABHR emerged in clinical practice in the 1980s [4, 11, 12]. The full search applied for MEDLINE (Additional file 1) was individualised for the other databases according to their functionality. Secondly, as keyword terms cannot be comprehensively combined in ScienceDirect, only the broadest “hand hygiene” search term was used for this database. Finally, the reference lists of included papers were searched manually to identify any additional relevant articles.

### Study selection

The titles and abstracts of all records identified in the search were screened for relevance against the eligibility criteria, with 43% of records screened by two independent reviewers (LG and LP or ES). The remaining 57% of records were screened by a single reviewer (LG), with uncertainties consulted with the second, experienced reviewer (LP). The full texts of articles that met the inclusion criteria after the title and abstract search, and those in which there was insufficient evidence in the title and abstract to make a decision, were reviewed in full text, with 55% of these records reviewed by two independent reviewers (LG and LP or ES), and 45% by a single reviewer (LG). At both stages of the study selection process, independent reviewers’ decisions were compared, and disagreements (e.g. when one reviewer judged the study to be eligible for inclusion, while another reviewer’s decision was to exclude) were identified and discussed between the independent reviewers with the aim of reaching consensus. If consensus could not be reached a third, experienced reviewer was asked to resolve the disagreement by reviewing the record and making a final decision.

### Quality assessment and data extraction

Two reviewers (LG and AJ, JCA or LP) independently extracted data from all studies included in the review, using a standardised data collection tool (Additional file 2). Extracted data included the study aim(s), country of origin, study settings, design, sample, intervention, intervention standardisation and fidelity, methods, study outcomes and relevant findings. Furthermore, full-text copies of all articles included in the review were independently reviewed by two reviewers (LG and AJ, JCA or LP) to assess their quality. All included studies met the Cochrane Effective Practice and Organization of Care (EPOC) criteria for study design, that is RCTs, NRTs, controlled before-and-after studies, interrupted time series studies or repeated measures studies [13]; and therefore, all included studies were assessed for quality using the recommended EPOC risk of bias criteria [14].

The content of data extraction and quality assessment spreadsheets, completed by two independent reviewers, were compared and inconsistencies were identified as disagreements. Disagreements were discussed between the independent reviewers with the aim of reaching a consensus, and if an agreed decision could not be reached a third opinion was sought from another reviewer who made the final decision.

### Analysis

The characteristics of the included studies were compared to assess for the appropriateness of conducting a pooled analysis. Due to the substantial heterogeneity of the studies, it was not appropriate to conduct a meta-analysis, nor to use the Grading of Recommendations Assessment, Development and Evaluation (GRADE) approach [15]. Instead, included studies were grouped into categories based on the focus of interest and further into sub-categories based on the outcome measure. The results were analysed with consideration of the quality assessment, study design, settings, data collection methods, and standardisation of the handrubbing procedure (i.e. ABHR products used, ABHR application technique, ABHR volume, application time and artificial contamination of the hands) and were synthesised in a narrative summary.

## Results

### Search results

**Fig. 1:**
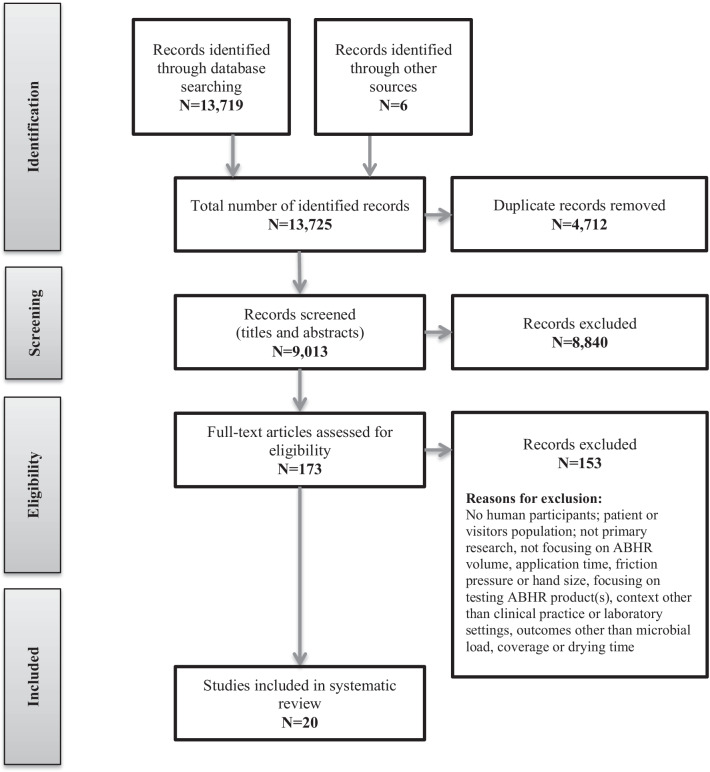
Study selection flowchart

The searches resulted in a total of 13,725 records. Of these, 4712 were duplicates, resulting in 9013 records being screened for eligibility. After screening of titles and abstracts, full texts of 173 articles were assessed, resulting in a total of 20 studies included in the review. The study selection process is shown in detail in Fig. 1.

All 20 studies met the EPOC criteria for study designs [13]. As shown in Tables 1, 2 and 3, this included two parallel design [16, 17], one crossover [18] and eight within-subject [19–26] RCTs, and one parallel design [27], one crossover [28] and seven within-subject [29–35] NRTs. Apart from one study [17] conducted in China, all included evidence derives from high income countries, including Australia (n = 1) [28], Austria (n = 2) [25, 33], France (n = 1) [31], Germany (n = 3) [16, 18, 32], Switzerland (n = 5) [23, 24, 26, 29, 30], UK (n = 3) [27, 34, 35] and USA (n = 2) [19, 22], while two were multisite studies conducted in the Netherlands and UK [21], and in Germany and USA [20]. Despite searching for studies published since 1980, the publication year of the included studies ranged from 2003 to 2020, with most studies (n = 15) published within the last 10 years. Furthermore, most studies (n = 15) were conducted in laboratory settings [19–26, 29–35], while five studies were based in a healthcare setting [16–18, 27, 28]. The study participants were HCWs [16–18, 21, 23, 24, 26–29, 31], and non-HCWs [20], while in eight laboratory-based studies participants were described as volunteers [19, 22, 25, 30, 32–35].

**Table 1:** Characteristics of studies investigating the influence of alcohol-based handrub volume

Author Date Country	Design	Setting	Participants	Tested ABHR volumes	Outcome measure(s)	Data collection method	Standardisation	Results
MacDonald et al (2006) [27] UK	NRT	Clinical	84 HCWs	1.75 ml 3.5 ml	Hand coverage	UV lightbox	*Product* ABHR gel containing fluorescent substance *Application time* no limit given *Application technique* rubbed hands as per their usual technique	*Mean missed areas (%)* 1.75 ml: 6.35% 3.5 ml: 1.23% (P < 0.001)
Kampf (2008) [19] USA	Within-subject RCT	Laboratory	16 volunteers	2.4 ml 3.6 ml	1) Bacterial load on hands; 2) Hand coverage	1) Glove juice; 2) Visually observed for sufficiency to cover all surfaces	*Product* 4 ABHRs—1. 85% ethanol, 2. 60% ethanol, 3. 62% ethanol, 4. 61% ethanol, reference 4% chlorhexidine; *Application time* rubbed hands until dry; *Application technique* covered all surfaces; *Artificial contamination* (*Serratia marcescens*)	*Mean log*_*10*_* reduction* (range for 4 ABHRs): 2.4 ml: 1.90–2.79 3.6 ml: 2.53–3.04 Volume had significant influence on the mean log_10_ reduction (P < 0.001) *% of subjects with sufficient coverage* of both hands 2.4 ml: 96.6% 3.6 ml: 93.6%
Rotter et al (2009) [25] Austria	Within-subject RCT	Laboratory	1) 15 volunteers 2) 5 volunteers	1ml 2 ml 3 ml	1) Bacterial load on hands; 2) Drying time	1) Fingertips; 2) Time measured until resistance noted	*Product* Liquid ABHR, 2-propanol 60% (v/v); *Application time* 15 s *Application technique* 6-steps; *Artificial contamination* (*E. coli*)	*Mean log RF (SD)* 1ml: 2.9 (0.6) 2 ml: 3.2 (0.7) 3 ml: 3.5 (0.6) Significant between 2 ml & 3 ml (P < 0.05); and between 1ml & 3 ml (P < 0.001) *Mean (SD) drying time (seconds)* 1ml: 23.4 (4.8) 2 ml: 35.0 (9.4) 3 ml: 49.4 (12.4)
Goroncy-Bermes, Koburger & Meyer (2010) [32] Germany	Within-subject NRT	Laboratory	16 volunteers	2 ml 2.5 ml 3 ml 4 ml	1) Bacterial load on hands; 2) Hand size; 3) Hand coverage	1) Fingertips; 2) Hand size calculated as hand length x width; 3) Sufficiency to cover all surfaces self-reported by participants	*Product* 2 ABHRs 1. 96% ethanol (46.0% w/w) + 2-propanol (27.0% w/w) + benzyl alcohol (1% w/w), 2. 96% ethanol (78.2% w/w) + 2-biphenylol (0.1% w/w), Reference liquid 2-propanol 60% (v/v); *Application time* 30 s; *Application technique* not explicitly stated; *Artificial contamination* (*E. coli*)	*Mean log*_*10*_* reduction* (for 2 tested ABHRs) 2 ml: 3.34 & 3.37 2.5 ml: 3.99 (only one ABHR tested) 3 ml: 3.94 & 4.47 4 ml: 4.19 & 4.52 For ABHR 1. 2 ml significantly lower reduction than for 3 ml (P = 0.009) or 4 ml (P ≤ 0.000) For ABHR 2. 2 ml significantly lower reduction than for 2.5 ml (P = 0.006), 3 ml (P = 0.001) or 4 ml (P = 0.001) *Hand size* Male hands significantly bigger than female hands (P < 0.001) No significant correlation between hand size and bacterial load reduction (P > 0.05) *Number of participants reporting applied volume as sufficient* to cover both hands (for 2 tested ABHRs) 2 ml: 7/16 & 4/16 3 ml: 14/15 & 15/15 4 ml: 14/15 (reported for one ABHR only)
Girard et al (2012) [31] France	Within-subject NRT	Laboratory	71 IPC workers	1.5 ml 2 ml 3 ml	Hand coverage	Sufficiency to cover all surfaces including the wrists (no details)	*Product* 27 ABHR named but contents not reported *Application technique* 6-steps + wrists	*Number (%) of cases in which applied volume was sufficient* 1.5 ml: 551/575 (95.8%) 2 ml: 530/538 (98.5%) 3 ml: 592/593 (99.8%)
Kampf et al (2013) [20] Germany/USA	Within-subject RCT	Laboratory	15 non-HCWs	1.1ml 2 ml 2.4 ml Single pump press Double pump press	1) Drying time; 2) Hand coverage	1) Time measured until participants reported that their hands felt dry; 2) UV light box	*Product* 3 ABHR—1. 85% (w/w) ethanol gel, 2. 70% (v/v) ethanol gel, 3. 70% (v/v) ethanol foam *Application technique* Covered all surfaces *Application time* rubbed until dry (for measuring hand coverage outcomes)	*Mean drying time (seconds)* 1.1ml: 20–25 2 ml: 37–41 2.4 ml: 41–49 Single press: 20–29 Double press: 34–53 *% of participants with incomplete coverage* 1.1ml: 67–87% 2 ml: 27–53% 2.4 ml: 13–27% Single press: 80–93% Double press: 0–47%
Li, XU & Zhao (2014) [17] China	RCT	Clinical	74 nurses	1.8 ml 3.6 ml	1) Bacterial load on hands; 2) Drying time	1) Imprint technique; 2) Time recorded until hands were dry	*Product* Gel ABHR; product contents not reported; *Application technique* 6-steps *Application time* not applicable (drying time measured as outcome)	*% reduction rate (SD)* 1.8 ml: 92.2% (10.8) 3.6 ml: 96.1% (5.6) (P = 0.049) *Drying time (seconds)* 1.8 ml: 44.1 3.6 ml: 75.3 (P < 0.001)
Macinga et al (2014) [22] USA	Within-subject RCT	Laboratory	13 volunteers	Different volumes (unspecified) of 6 ABHRs	Drying time	Time measured until participants reported that their hands felt dry	*Product* 6 ABHRs 1. 62% (v/v) ethanol foam; 2. 70% (v/v) ethanol foam; 3. 70% (v/v) ethanol gel; 4. 90% (v/v) ethanol gel; 5. 70% (v/v) ethanol rinse; 6. 80% (v/v) ethanol rinse *Application technique* rubbed hands until dry	ABHR volumes indicated to dry in 30 s ranged from 1.7 to 2.1ml Drying rate (seconds/ml) ranged from 12.2 (95% CI, 9.8–14.7) to 18.2 (95% CI, 15.5–21.0)
Bellissimo-Rodrigues et al (2015) [29] Switzerland	Within-subject NRT	Laboratory	15 HCWs	0.5 ml to 3 ml, in 0.5 ml variations (with addition of 4, 5, and 6 ml for large hands)	1) Bacterial load on hands; 2) Hand size	1) Fingertips; 2) Hand surface area calculation Small < 375cm^2^ Medium 376–424 cm^2^ Large > 425 cm^2^	*Product* liquid ABHR, 2-propanol 60%; *Application time* 30 s; *Application technique* 6-steps; *Artificial contamination* (*E. coli*)	The *mean bacterial reduction* was 0.28 log_10_ for each additional 0.5 ml of ABHR (95% CI, 0.20–0.36; P < 0.001) *Hand size* Mean log_10_ reduction per each additional 0.5 ml of ABHR: Small hands 0.40 (95% CI, 0.27–0.52; P < 0.001) Medium hands 0.32 (95% CI, 0.21–0.42; P < 0.001) Large hands 0.15 (95% CI, 0.03–0.26; P = 0.011) Bacterial reduction was inversely and significantly associated with hand surface area (− 0.003 [95% CI, − 0.006– − 0.0005], P = 0.019)
Wilkinson et al (2017) [34] UK	Within-subject NRT	Laboratory	1) 5 volunteers 2) 15 volunteers 3) 15 volunteers	0.5 ml to 3 ml, in 0.5 ml variations	1) Bacterial load on hands; 2) Hand size; 3) Drying time	1) Fingertips; 2) Hand surface area = 2.48 × hand length x hand breadth; 3) Time measured until participants reported that their hands felt dry	*Product* 3 ABHRs—1. ethanol, 80% (v/v); glycerol, 1.45% (v/v); hydrogen peroxide, 0.125% (v/v) 2. isopropyl alcohol, 75% (v/v); glycerol, 1.45% (v/v); hydrogen peroxide, 0.125% (v/v) 3. Reference liquid 60% IPA: isopropyl alcohol, 60% (v/v); *Application time* 30 s (except when volumes were tested for drying times) *Application technique* 6-steps; *Artificial contamination* (*E. coli*)	*Log*_*10*_* RF* (for 3 tested ABHRs): 0.5 ml: 2.15–2.80 1ml: 2.22–2.98 1.5 ml: 2.82–3.08 2 ml: 3.22–3.81 2.5 ml: 3.80–4.27 3 ml: 3.91–4.60 Significant correlation between volume and bacterial reduction (P < 0.001) *Hand size* Log_10_ RF was not significantly associated with hand size (P = 0.9782) *Drying times (seconds)*, (for 3 tested ABHRs) 0.5 ml: 10.07–11.40 1ml: 16.33–17.53 1.5 ml: 20.73–27.07 2 ml: 26.93–31.00 2.5 ml: 33.13–37.20 3 ml: 36.60–45.73 Drying time had a significant, positive association with volume (P < 0.001)
Wilkinson et al (2018) [35] UK	Within-subject NRT	Laboratory	15 volunteers	0.5 ml to 3 ml, in 0.5 ml variations	Drying time	Time measured until participants reported that their hands felt dry	*Product* 2 ABHRs—1. 60% (v/v) isopropanol; 2. 80% (v/v) ethanol + 1.45% (v/v) glycerol + 0.125% (v/v) hydrogen peroxide (each in liquid, gel & foam format) *Application technique* 6-steps;	*Mean drying times (seconds)*, for 2 tested ABHRs in 3 formats) 1.5 ml: 19.67–31.53 3 ml: 35.07–63.13 Increasing the volume increased the drying time (P < 0.001)
Jain, Clezy & McLaws (2018) [28] Australia	Crossover NRT	Clinical	40 HCWs	2 dispenser pump presses vs. 3 dispenser pump presses	Bacterial load on hands	Fingertips (cultured for MRSA and VRE)	*Product* foam ABHR; product contents not reported *Application time* rubbed until hands were dry *Application technique* 6-steps	*Number of plates* with MRSA or VRE growth: 2 dispenser pumps: 2/40 grew 1 colony-forming unit of MRSA 3 dispenser pumps: No growth
Suchomel et al (2018) [33] Austria	Within-subject NRT	Laboratory	15 volunteers	1ml 2 ml 3 ml	1) Bacterial load on hands; 2) Hand size; 3) Drying time	1) Fingertips; 2) Hand surface area calculation Small < 375cm^2^ Medium 376–424 cm^2^ Large > 425 cm^2^; 3) Time measured until participants reported that their hands felt dry	*Product* ABHR 2-propanol 60% (v/v) liquid; *Application time* not applicable (drying time measured as outcome) *Application technique* 6-steps *Artificial contamination* (*E. coli*)	*Mean log*_*10*_* RF (SD)* 1ml: 1.99 (0.66) 2 ml: 2.96 (0.84) 3 ml: 3.28 (0.96) Mean log_10_ RFs were greater when larger volumes were used (P < 0.0001), but no significant difference between 2 ml & 3 ml (P = 0.08) *Hand size* and log_10_ RF (P = 0.698). Hand size and RF accounting for dry-times and volumes (R^2^ = 77%, P = 0.403) *Mean (SD) drying (seconds)* 1ml 24 (7) 2 ml 50 (14) 3 ml 67 (20) (P ≤ 0.030) Mean drying times were greater when larger application volumes were used (P < 0.0001) Regardless of volume Reduction fraction increased 0.29 log_10_ per 10 s increased drying time
Kenters et al (2020) [21] Netherlands	Within-subject RCT	Laboratory	1) 9 HCWs; 2) 10 HCWs	0.75 ml 1.5 ml 2.25 ml 3 ml	1) Drying time; 2) Hand coverage	1) Time measured until participants reported that their hands felt dry; 2) Hands photographed under UV light	*Product* 65% ethanol, + 10% n-propanol (in gel, foam & liquid formats). For hand coverage outcome—ABHR in gel & foam format mixed with a 2% concentration UV marker; product contents not reported *Application time* rubbed until dry (for hand coverage outcome) *Application technique* 6-steps	0.75, 1.5 & 2.25 ml dried within 20–30 s 3 ml dried between 37 and 56 s *Hand coverage* At least 2.25 ml required for optimal coverage. Foam covered 90% & gel 82% hands

*ABHR* alcohol-based handrub, *CI* confidence intervals, *E. coli Escherichia coli*, *HCW* healthcare workers, *MRSA* Meticillin-resistant *Staphylococcus aureus*, *NRT* non-randomised trial, *RCT* randomised controlled trial, *RF* reduction factor, *SD* standard deviation, *UV* ultraviolet, *VRE* vancomycin-resistant enterococci

**Table 2:** Characteristics of studies investigating the influence of alcohol-based handrub application time

Author Date Country	Design	Setting	Participants	Tested ABHR application times	Outcome measure(s)	Data collection method	Standardisation	Results
Dharan et al (2003) [30] Switzerland	Within-subject NRT	Laboratory	12 volunteers	15 s 30 s	Bacterial load on hands	Fingertips	*Product* 4 ABHR—1. 80% ethanol (w/v) rinse; 2. 95% ethanol (w/v) rinse; 3. 75% isopropanol (v/v) & 0.5% chlorhexidine rinse 4. 60% isopropanol gel Reference 2-propanol 60% (v/v) *ABHR volume* 3 ml *Application technique* ABHR was applied to the cupped fingertips of the right hand, which were disinfected by rubbing of the thumb against fingertips and fingernails *Artificial contamination* (*S. aureus*, *Pseudomonas aeruginosa*, *Enterococcus faecalis*)	*Mean log*_*10*_* RF* at 30 s significantly higher than 15 s for all ABHR products (P < 0.01)
Rotter et al (2009) [25] Austria	Within-subject RCT	Laboratory	15 volunteers	15 s 30 s 60 s	Bacterial load on hands	Fingertips	*Product* liquid ABHR 2-propanol 60% (v/v) *ABHR volume* 3 ml *Application technique* 6-steps *Artificial contamination* (*E. coli*)	*Mean log RF (SD)* 15 s: 3.5 (0.8) 30 s: 3.7 (0.8) 60 s: 4.5 (0.8) Significant between 30 s & 60 s (*P* < 0.01); and between 15 s & 60 s (*P* < 0.001)
Kramer et al (2017) [16] Germany	RCT	Clinical	14 nurses	15 s 30 s	Bacterial load on hands	Fingertips	*Product* ABHR 45% (w/w) propan-2-ol + 30% (w/w) propan-1-ol + 0.2% mecetroniumetile sulfate *Average ABHR volume used* 3.4 ml *Application technique* 6-steps	*Mean log RF (SD)* 15 s: 1.24 (0.68) 30 s: 1.31 (0.61) (P = 0.59)
Pires et al (2017) [24] Switzerland	Within-subject RCT	Laboratory	1) 23 HCWs 2) 18 HCWs	1) 10 s 15 s 20 s 30 s 45 s 60 s 2) 15 s 30 s	Bacterial load on hands	Fingertips	*Product* ABHR isopropanol 60% (v/v) *ABHR volume* 3ml *Application technique* 6-steps *Artificial contamination* (*E. coli*)	*Mean log*_*10*_* reduction* after 15 s non-inferior to 30 s; 0.11 log_10_ lower (95% CI, − 0.46–0.24) All durations resulted in a reduction in bacterial count (P < 0.001) Reductions after 10, 15 or 20 s not different to 30 s (P = 0.174, 0.312, 0.720) Reductions after 30 s higher than 45 or 60 s (P = 0.004, 0.011)
Pires et al (2019) [23] Switzerland	Within-subject RCT	Laboratory	18 HCWs	15 s 30 s	Bacterial load on hands	Fingertips	*Product* ABHR isopropanol 60% (v/v) *ABHR volume* customized to hand size Small hands: 2.2 ml (IQR 2.2–2.4); Medium hands: 2.3 ml (IQR 2.2–2.3); Large hands: 3.2 ml (IQR 3.0–3.4) *Application technique* variations 6-steps according to daily routine *Artificial contamination* (*E. coli* & *S. aureus*)	*Log*_*10*_* RF* 15 s non-inferior to 30 s -0.06 log_10_ (95% CI, − 0.34–0.22; *P* = 0.659)
Harnoss et al (2020) [18] Germany	Crossover RCT	Clinical	14 nurses	15 s 30 s	Bacterial load on hands	Fingertips	*Product* liquid ABHR 45% (w/w) propan-2-ol + 30% (w/w) propan-1-ol + 0.2% mecetroniumetile *ABHR volume* 4 ml *Application technique* 6-steps	*Mean log*_*10*_* RF (SD)* 15 s: 0.92 (0.47) 30 s: 0.89 (0.45) (*P* = 0.638)

ABHR – alcohol-based handrub; CI – confidence intervals; *E. coli* – *Escherichia coli*; HCW – healthcare workers; *S. aureus* – *Staphylococcus aureus*; IQR – interquartile range; NRT – non-randomised trial; RCT – randomised controlled trial; RF – reduction factor; SD – standard deviation

**Table 3:** Characteristics of the study investigating the influence of rubbing friction

Author Date Country	Design	Setting	Participants	Comparators	Outcome measure(s)	Data collection method	Standardisation	Results
Tan et al (2020) [26] Switzerland	Within-subject RCT	Laboratory	19 HCWs	1) Poured ABHR + rubbing; 2) Sprayed ABHR + rubbing; 3) Sprayed ABHR without rubbing	Bacterial load on hands	Fingertips	*Product* ABHR isopropanol 60% (v/v) *Application time* 30 s *ABHR volume* 3 ml *Application technique* 6-step (for intervention 1 & 2); sprayed ABHR without hand rubbing (for intervention 3) *Artificial contamination* (*E. coli*)	*Mean log*_*10*_* reduction (95% CI)* Poured ABHR + rubbing: 3.46 (1.27–5.65); sprayed ABHR + rubbing: 3.66 (1.68–5.64); sprayed ABHR without rubbing: 2.76 (1.65–3.87) Sprayed ABHR without hand rubbing resulted in significantly lower bacterial load reduction than poured or sprayed ABHR with hand rubbing (− 0.70; 95% CI: − 1.13 to − 0.28)

*ABHR* alcohol-based handrub, *CI* confidence intervals, *E. coli* *Escherichia coli*, *HCW* healthcare workers, *RCT* randomised controlled trial

Studies were grouped into three categories, based on the focus of interest. These included: Volume of ABHR (n = 14), [17, 19–22, 25, 27–29, 31–35] Application time of ABHR (n = 6) [16, 18, 23–25, 30] and Rubbing friction (n = 1) [26]. One of the studies [25] was included in both Volume of ABHR and Application time of ABHR categories because it reported a series of experiments, of which one investigated the impact of shortening handrubbing duration and another one the impact of ABHR volume. None of the included studies investigated hand size as an area of focus; however, four studies that focused on the influence of ABHR volume on bacterial load reduction additionally accounted for participants’ hand size in their analysis [29, 32–34]. The studies within the Volume of ABHR category were further grouped into four sub-categories, based on the outcome measure, including the sub-category of studies that accounted for hand size. Therefore, the sub-categories included the influence of ABHR volume on: bacterial load reduction (n = 8) [17, 19, 25, 28, 29, 32–34]; bacterial load reduction with consideration of hand size (n = 4) [29, 32–34]; drying time (n = 8) [17, 20–22, 25, 33–35] and hand surface coverage (n = 6) [19–21, 27, 31, 32]. Furthermore, most studies included in the Volume of ABHR category focused on more than one outcome measure. Thus, findings in relation to each outcome are discussed separately for each outcome measure sub-category. Finally, all studies within the Application time of ABHR and within the Rubbing friction categories had outcomes related to bacterial load reduction.

### Volume of ABHR

#### Influence of ABHR volume on bacterial load reduction

As shown in Table 1, six laboratory-based studies, including two within-subject RCTs [19, 25], four within-subject NRTs [29, 32–34] and two clinically based studies, including an RCT [17] and a crossover NRT [28], investigated the influence of ABHR volume on bacterial load reduction as an outcome. Tested ABHR volumes ranged from 0.5 ml to 6 ml, and studies varied in terms of the methods used and intervention standardisation.

Apart from Kampf (2008) [19], who used the glove juice technique to collect samples from the entire surface of the hands, all other studies collected samples using the fingertip method [25, 28, 29, 32–34] or imprint method [17]; however, the imprinted areas of hand were not specified in Li et al. [17]. In six laboratory-based studies, participants’ hands were artificially contaminated, whereas in clinically based studies, the reduction in the bacterial load naturally present on participants’ hands was measured. Sample size in laboratory-based studies ranged from 5 to 16, and each one described their sample as volunteers, apart from Bellissimo-Rodrigues et al. (2015) [29] in which the sample was HCWs. In the clinical studies, 74 nurses [17] and 40 HCWs [28] were involved. The 6-step technique was used for the application of ABHR in six studies [17, 25, 28, 29, 33, 34]. In one study, participants covered all hand surfaces [19], while Goroncy-Bermes, Koburger & Meyer (2010) [32] did not clearly specify what technique was used. In four studies, application time was standardised to 15 [25] or 30 s [29, 32, 34]. In two studies [19, 28], participants rubbed hands until dry. In another two studies [17, 33], ABHR drying time was measured as an outcome in addition to bacterial load on hands. Finally, the ABHR products used differed across the studies in terms of the format (i.e. liquid, gel or foam), formulation and concentration, with three studies investigating the volumes of two [32], three [34] or four [19] different ABHR products.

Findings were consistent in demonstrating that as the ABHR volume increased, bacterial reduction also increased. Three laboratory-based studies demonstrated a significant, positive relationship between ABHR volume and mean log_10_ reduction in bacterial load on hands (*P* < 0.001) [19, 29, 34], with Bellissimo-Rodrigues et al. (2015) [29] reporting a 0.28 log_10_ increase in the mean bacterial load reduction on hands for each additional 0.5 ml of ABHR (*P* < 0.001). Another two laboratory studies compared different volumes of ABHR [25, 32]. One demonstrated that 3 ml volume resulted in significantly greater mean log_10_ reduction factors (RF) when compared to 2 ml (*P* < 0.05) or 1ml (*P* < 0.001) volumes [25], while in the second study, 2 ml volume resulted in significantly lower mean log_10_ reduction in comparison to 2.5 ml (*P* = 0.006), 3 ml (*P* < 0.01) or 4 ml (*P* ≤ 0.001) [32]. Finally, Suchomel et al. [33], who compared 1 ml, 2 ml and 3 ml volumes reported that the mean log_10_ reduction was significantly greater when larger volumes were used (*P* < 0.0001); however, the authors found no significant difference between 2 and 3 ml volumes (*P* = 0.08).

Both clinically based studies compared two volumes of ABHR. Li et al. [17] demonstrated that 3.6 ml volume resulted in significantly greater bacterial load reduction on hands when compared to 1.8 ml volume (*P* = 0.049). Jain et al. [28] compared ABHR volume delivered by double and by triple dispenser pump presses for the presence of methicillin-resistant *Staphylococcus aureus* (MRSA) or vancomycin-resistant enterococci growth in the samples after hand rubbing and reported that using double pump press volume resulted in MRSA growth in two out of 40 samples, in comparison to none of the samples when triple pump press volume was used.

#### Influence of ABHR volume on bacterial load reduction with consideration of hand size

Four of the aforementioned laboratory-based within-subject NRTs that investigated the influence of ABHR volume on bacterial load reduction also considered participants’ hand size in their analysis [29, 32–34]. In four studies, hand size was determined by calculating hand surface area. Both Wilkinson et al. (2017) [34] and Bellissimo-Rodrigues et al. (2015) [29] used the formula described by Hsu and Yu (2010) [36]: 2.48 × hand length x hand breadth, Suchomel et al. (2018) [33] the formula recommended by Lee, Choi & Kim (2007) [37]: 1.219 × hand length x hand circumference, while Goroncy-Bermes, Koburger & Meyer (2010) [32] multiplied hand length by its width. Furthermore, three studies [29, 33, 34] used regression analysis to examine the relationship between ABHR volume, bacterial load reduction and hand size, while Goroncy-Bermes, Koburger & Meyer (2010) [32] investigated for the correlation between the hand size and microbial load reduction for each of the tested ABHR volumes using regression analysis in addition to investigating for the difference in bacterial load reduction between small female hands and large male hands per ABHR volume.

Findings on the influence of hand size on bacterial load reduction were inconsistent. Bellissimo-Rodrigues et al. (2015) [29] demonstrated a significant, negative association between hand size and bacterial load reduction (− 0.003 [95% CI, − 0.006–− 0.0005], *P* = 0.019) and reported the mean log_10_ reduction per each additional 0.5 ml of ABHR was 0.40 (95% CI, 0.27–0.52, *P* < 0.001) for small hands, 0.32 (0.21–0.42, *P* < 0.001) for medium hands, and 0.15 (0.03–0.26, *P* = 0.011) for large hands. On the contrary, the remaining three studies found no significant relationship between hand size and bacterial load reduction (*P* < 0.05; [32] *P* = 0.698 [33]; *P* = 0.978 [34]). However, in Goroncy-Bermes, Koburger and Meyer [32] plateau levels where no further increase in bacterial load reduction could be achieved by applying greater volumes of ABHR were reached on smaller, female hands with lower amounts of product (2.5–3 ml) in comparison to larger, male hands (≥ 3 ml).

#### Influence of ABHR volume on ABHR drying time

Seven laboratory-based studies, including four within-subject RCTs [20–22, 25], three within-subject NRTs [33–35] and one clinically based RCT [17] investigated the influence of ABHR volume on drying time outcomes (Table 1). Tested ABHR volumes, methods used to collect drying time data and aspects of intervention standardisation differed across the studies. Tested volumes ranged from 0.5 to 3.6 ml. In the laboratory-based studies, sample size ranged from 5 to 15, with all describing their participants as “volunteers”, “subjects”, or non-HCWs, with the exception of Kenters et al. (2020) [21] study in which participants were HCWs, whereas the clinically based study by Li et al. (2014) [17] involved 74 nurses. Apart from Li et al. (2014) [17], who provided no details on how drying time was measured, in all studies time was measured until participants reported that their hands felt dry [20–22, 33–35] or until resistance was noted while rubbing the hands together [25].

In six studies [17, 21, 25, 33–35] the ABHR technique was standardised to 6-steps. In one study [20] participants used “responsible application” which involves covering all hand surfaces with ABHR without following any particular steps, while Macinga et al. (2014) [22] made no mention of providing any specific instructions to the participants about how to apply ABHR. A variety of ABHR products were used across the studies, differing in the format, formulation and concentration, with five studies [20–22, 34, 35] investigating the volumes of more than one ABHR formulation or format.

Similarly to the influence of volume on bacterial load reduction, with regards to the drying time outcome, evidence is mostly consistent in that as the ABHR volume increased, drying time also increased; however in Rotter et al. (2009) [25] and Kampf et al. (2013) [20] this evidence was based on descriptive results, with levels of significance not provided. Two laboratory-based studies [34, 35] demonstrated a significant, positive association between ABHR volume and drying time (*P* < 0.001). Meanwhile, a third study [33] reported that while the mean drying times were greater when larger application volumes were used (*P* < 0.0001), ABHR volume did not have a statistically significant effect in addition to dry-times (*P* = 0.172) and log_10_ RF increased 0.29 for every 10 s increase of drying time, regardless of the volume of ABHR. This suggests that the drying time, rather than ABHR volume was the key driver of efficacy [33].

Macinga et al. (2014) [22] and Kenters et al. (2020) [21] used a different approach as they aimed to identify ABHR volumes required to dry in 20–30 s. The former showed that volumes ranging from 1.7 to 2.1 were required to achieve 30 s drying time and drying rate ranged from 12.2 s/ml (95% CI, 9.8–14.7) to 18.2 (95% CI, 15.5–21.0) [22], while the latter reported that 0.75 ml, 1.5 ml and 2.25 ml volumes of ABHR all dried within 20–30 s [21].

Finally, the findings from a clinically based study by Li et al. (2014) [17] indicated that using 3.6 ml of ABHR resulted in significantly longer drying time in comparison with 1.8 ml volume (*P* < 0.001).

#### Influence of ABHR volume on hand surface coverage

Of the studies focusing on ABHR volume, six used hand surface coverage as an outcome, including five laboratory-based within-subject RCT [19, 20] or NRTs [21, 31, 32], and one clinically based NRT [27]. The range of ABHR volumes tested in these studies was between 0.75 ml and 4 ml. In three studies [20, 21, 27] hand surface coverage was determined using ultraviolet light source after participants applied specific volume of fluorescent ABHR, while in three studies coverage was assessed by the participants or an investigator as sufficient to cover all hand surfaces [19, 31, 32]. In laboratory-based studies, participants were volunteers [19, 32], HCWs [21, 31] or non-HCWs [20], with sample size ranging from 10 to 71, while the clinically based study sample consisted of 84 HCWs.

In two laboratory-based studies participants used either a standard 6-step technique [21], or 6-steps with an additional step involving rubbing of the wrists [31]. In Kampf (2008) [19] and Kampf et al. (2013) [20], participants applied ABHR to cover all hand surfaces, without any specific instructions followed, while Goroncy-Bermes, Koburger & Meyer (2010) [32] did not specify the application technique in their instructions. Apart from Goroncy-Bermes, Koburger & Meyer (2010) [32] who standardised the duration of hand rubbing to 30 s, in all laboratory-based studies participants were asked to rub their hands until dry. Furthermore, in all laboratory-based studies, the volumes of more than one ABHR formulation or format were used, with a range from two to 27 different ABHR products. In the clinically based study by MacDonald et al. (2006) [27] participants were asked to rub their hands as they normally would, using the same fluorescent ABHR, with no restrictions to the hand rubbing duration.

All laboratory-based studies provided only descriptive findings on the influence of volume on hand surface coverage, with the levels of significance not reported. Three studies showed that the number of cases in which applied ABHR volume was assessed to be sufficient to cover all hand surfaces [31, 32], or the number of participants with complete coverage [20] was greater when larger ABHR volumes were used. However, in one study [19] the percent of participants with sufficient hand coverage was slightly greater (96.6%) after application of 2.4 ml volume of ABHR, than when 3.6 ml were applied (93.6%, P-value not reported). Yet another study [21] identified that at least 2.25 ml of ABHR were required to achieve optimal hand coverage, with ABHR foam applied at this volume resulting in an average 90% coverage while ABHR gel achieved 82%. The only statistically significant finding derived from a clinically based study [27] demonstrated that the mean percentage of missed areas was significantly greater when 1.75 ml volume of ABHR was used (6.35%), in comparison with the larger 3.5 ml volume (1.23%; P < 0.001).

#### Volume of ABHR: summary

With regards to the studies investigating the influence of ABHR volume on bacterial load reduction, five out of six studies demonstrated either a significant, positive relationship between ABHR volume and bacterial load reduction on hands or a significant difference in bacterial reduction between different volumes, in favour of ABHR volumes that were equal to or larger than 3 ml. Consistent agreement was also found amongst studies that investigated the influence of ABHR volume on drying time, with five laboratory-based within-subject trials and one clinically based RCT demonstrating that as ABHR volume increased, drying time also increased. Another two laboratory-based within-subject RCT showed that ABHR volumes between 1.7 and 2.25 ml were required to dry within 20–30 s, while one laboratory-based within-subject NRT indicated that drying time, rather than ABHR volume was the prime driver of efficacy. However, the evidence on the influence of ABHR volume on hand size is inconsistent, with three laboratory-based within-subject NRTs showing the lack of a relationship and another one demonstrating a significant association between hand size and bacterial load reduction when fixed ABHR volumes were used.

With respect to the hand surface coverage outcome, apart from one laboratory-based study, evidence was consistent in reporting that the largest ABHR volumes tested resulted in the highest hand surface coverage, with one study reporting that at least 2.5 ml of ABHR was required to achieve 82–90% coverage. However, this evidence is mainly based on descriptive findings, and only one, clinically based study demonstrated a significant difference in hand coverage between 1.75 and 3.5 ml volume, in favour of the larger volume.

### Application time of ABHR

#### Influence of ABHR application time on bacterial load reduction

The influence of ABHR application time on bacterial load reduction was the focus of six studies. These included three laboratory-based within-subject RCTs [23–25], one laboratory-based within-subject NRT [30], and two clinically based parallel [16] or crossover RCTs [18] (Table 2). All six studies compared 15 s and 30 s application times but in addition, Rotter et al. (2009) [25] also compared these to 60 s, while in a separate experiment, Pires et al. (2017) [24] also compared six different application durations, ranging from 10 to 60 s. However, methods, ABHR volumes, application technique and products varied across the studies.

In the laboratory-based experiments, participants were described as volunteers [25, 30] or HCWs [23, 24] with sample size range of 12–23, while each of the two clinical studies involved 14 nurses [16, 18]. In all six studies, samples were collected from participants’ hands using the fingertip method, with all four laboratory-based studies involving artificial contamination of participants’ hands. With regards to the intervention standardisation, in four studies [16, 18, 24, 25], the 6-step technique was used by the participants to apply ABHR to hands. In Dharan et al. (2003) [30], ABHR was applied to the cupped fingertips of the right hand, which were cleaned by rubbing of the thumb against fingertips and fingernails, while Pires et al. [23] stated that participants performed variations of the 6-step technique according to their daily routine, but no specific advice was given. In four studies, the volume of ABHR was standardised to 3 ml [24, 25, 30] or 4 ml [18], while in one study [23], ABHR volume was customized to hand size. In Kramer et al. [16] study, ABHR volume was monitored with an average of 3.4 ml being used. Regarding ABHR product, apart from Dharan et al. (2003) [30] who tested four different ABHR formulations, in all studies single ABHR product was used for all tests.

With regards to 15 s versus 30 s application times, evidence was inconsistent. Two laboratory-based RCTs [23, 24] showed that 15 s application time was non-inferior to 30 s (0.11 log_10_ lower; 95% CI, − 0.46–0.24 [24] and − 0.06 log_10_; 95% CI, − 0.34–0.22; *P* = 0.659 [23]). Furthermore, two clinically based RCTs showed no significant difference between 15 and 30 s (*P* = 0.59 [16] and *P* = 0.64 [18]). However, one laboratory-based NRT [30] demonstrated that the reduction in bacterial load was significantly higher at 30 s application time in comparison with 15 s (*P* < 0.01). Yet another laboratory-based RCT [25] compared 15 s, 30 s and 60 s application times and found that bacterial load reduction increased as the application time increased, but the difference was significant between 30 and 60 s (*P* < 0.01) and between 15 and 60 s (*P* < 0.001), in favour of the longer application time. In addition, Pires et al. (2017) [24] compared six different application times ranging from 10 to 60 s and found that microbial load reduction achieved after 10, 15 and 20 s did not significantly differ from reduction achieved after 30 s. Interestingly, 30 s application time resulted in significantly higher microbial load reduction when compared to 45 s (P = 0.004) or 60 s (P = 0.011) [24].

#### Application time of ABHR: summary

Both clinically based studies showed no significant difference between the two application times (15 or 30 s). Furthermore, in the three out of four laboratory-based studies only one NRT showed significant superiority of 30 s application time in comparison with 15 s, while the remaining three studies found no significant difference between the two application times or demonstrated that 15 s was non-inferior to 30 s. With regards to longer application times, further inconsistencies were found across studies. One laboratory-based RCT found that microbial load reduction after 60 s application of ABHR was significantly greater in comparison with 15 or 30 s, and another showed that microbial load reduction was significantly lower for both 45- and 60-s application times in comparison with 30 s.

### Rubbing friction

#### Influence of rubbing friction on bacterial load reduction

As shown in Table 3, only one study [26] investigated the influence of rubbing friction on bacterial load reduction. It was a laboratory-based within-subject RCT that compared three protocols amongst 19 HCWs. These included: (1) hand rubbing using the 6-step technique, for 30 s using 3 ml of ABHR poured on to a palm of the hand, (2) hand rubbing using the 6-step technique, for 30 s using 3 ml of ABHR sprayed onto hands and (3) using 3 ml of ABHR sprayed onto hands, without hand rubbing and with hands held in an uprights position without moving for 30 s after applying ABHR to let the hands dry [26]. A single, ABHR product was used for all tests [26]. To determine bacterial load reduction, participants’ hands were artificially contaminated and samples were collected from their fingertips before and after application of ABHR [26]. The findings showed that using ABHR spray without hand rubbing resulted in significantly lower bacterial load reduction than using poured or sprayed ABHR with rubbing (− 0.70; 95% CI, − 1.13–− 0.28).

#### Rubbing friction: summary

The only study that investigated the influence of the rubbing friction on bacterial load reduction showed that the application of sprayed ABHR without hand rubbing was inferior to the use of ABHR with rubbing.

### Methodological quality of included studies

All studies included in the review used an experimental or quasi-experimental design and all met the EPOC criteria for study design [13]. However, there was a substantial heterogeneity across the studies with respect to the interventions, intervention standardisation, data collection methods and outcomes. Furthermore, when assessed using the EPOC standard risk of bias criteria [14], all of the included studies had at least one item assessed as high risk; thus, the overall risk of bias of all included studies was assessed as high (Additional file 3).

High risk of bias was associated with the lack of random assignment in NRT studies [21, 28, 29, 31–35]. Furthermore, most of the RCTs provided insufficient information on the generation of sequence allocation [16, 17, 19, 20, 22, 24–26], leading to unclear risk. Only two RCTs [18, 23] clearly described the sequence generation process using computer-generated random sequence to allocate participants to study arms, thereby reducing selection bias. However, with regards to the allocation concealment, all included studies were assessed as being of high risk, with the exception of two RCTs [16, 17] for which this risk was assessed as unclear. Thus, in all studies there was a degree of risk that assignment could be foreseen.

Baseline outcome data was only collected in six studies [16–19, 23, 30] and no significant differences were reported across the groups or identified differences were appropriately accounted for in the analysis. The remaining studies did not measure outcomes at baseline or provided insufficient details. With regards to participants’ baseline characteristics, these were not mentioned in two parallel RCTs [16, 17]; thus, there is a potential risk that there were significant differences between the groups. In addition, one multisite within-subject NRT [31] did not provide details on participants’ characteristics at each test centre; thus, it is unclear whether there were differences in sample characteristics between the sites. The remaining studies either clearly reported participant characteristics being similar across the groups [27] or used a crossover [18] or within-subject design [19–26, 28–30, 32–35] in which the same participants were involved in all study arms; thus, baseline characteristics can be judged to be similar.

One study [24] was assessed as being of high risk of bias associated with incomplete outcome data because of a marked imbalance in the proportion of missing data across the study arms, while for 11 studies [16, 17, 19, 21–23, 26, 31, 32, 34, 35] there was insufficient reporting of attrition to make a clear judgement on the risk of bias resulting from missing data, leading to an unclear risk. In the remaining nine studies [18, 20, 25, 27–30, 33], there were no missing outcome measures; thus, unlikely to bias the results.

Only three studies reported measures to prevent the knowledge of the allocation during the study by blinding data collectors [20, 35] or investigators who processed and quantified bacterial samples [23]. Furthermore, MacDonald et al. (2006) [27], Girard et al. (2012) [31] and Harnoss et al. (2020) [18] were open-label trials, which creates a risk of detection bias and affects the robustness. The remaining studies did not provide sufficient details to judge whether knowledge of the allocated interventions was adequately prevented during the study; thus, it is difficult to assess the reliability of the data collection process in these studies.

Another common risk of bias was contamination either resulting from allocation at the individual level or from a crossover or within-subject designs in which assignments were reversed or rotated; thus, could be predicted. Only one multisite study [31] was assessed as being of low risk of contamination, which resulted from the order of interventions being assigned independently in each test centre. In addition, in two parallel design studies [18, 27] allocation was by ward within a single department or hospital; thus, communication between the groups could occur and therefore, the risk of contamination was assessed as unclear.

Finally, apart from three studies for which reporting of results was insufficient [21, 22], or some of the outcomes stated in the methods section were not reported in the results [17], the risk of selective outcome reporting was assessed as low for all studies. However, some other potential biases, not included in the EPOC risk of bias criteria [14] were also identified for all of these studies.

Firstly, none of the studies report conducting a priori power analysis; thus, it is unclear whether their sample size was adequate. Furthermore, of the studies evaluating microbial load on hands, only one [19] used glove juice sampling method, which allows recovery of the bacterial load present on the entire surface of the hand [38] while the remaining studies relied on the fingertip kneading or imprint techniques, which reflect the bacterial load present only on the sampled part of the hand. Furthermore, all laboratory-based studies used artificial contamination of participants’ hands that standardised baseline bacterial load. However, such artificial contamination does not reflect the natural conditions and the actual bacterial flora present on HCWs’ hands. Of the studies that evaluated hand surface coverage outcome, three [20, 21, 27] used fluorescent ABHR and ultraviolet light, while in the remaining three studies [19, 31, 32] more subjective assessment of “sufficiency” of applied ABHR to cover all hand surfaces was used. Drying time outcomes were also assessed using rather subjective methods involving measuring time until hands felt dry [17, 20–22, 33–35] or until marked resistance was noted while rubbing hands together, and none of these studies reported inter- or intra-rater variability [25]; which limits validity and reliability of the measurements.

## Discussion

Hand rubbing with ABHR is commonly used in healthcare settings across the world and has been shown to impact on healthcare-associated infection outcomes [39, 40]; yet the evidence with respect to the factors which impact on its effectiveness is not well described. To our knowledge, this systematic review is the first to evaluate evidence on factors influencing the effectiveness of hand rubbing with ABHR, including ABHR volume, application time, hand size and rubbing friction.

With regards to the volume of ABHR, evidence consistently showed bacterial load reduction on hands and ABHR drying time both increase with greater ABHR volumes. However, volumes as small as 1–2 ml were found sufficient [17, 20, 21, 25, 33–35] to keep both hands wet for the 20–30 s recommended in the guidelines [2, 5]; however, some of the reviewed evidence demonstrated that application of such small volumes resulted in suboptimal hand surface coverage [20, 21, 32] and more importantly, in significantly smaller bacterial load reduction, in comparison with larger volumes [17, 25, 32].

Simply increasing recommended volume is not justifiable at this stage, not only from the lack of evidence but greater volumes may not be acceptable to healthcare staff. Volumes equal to or greater than 2.4 ml were consistently reported to require longer than 30 s to dry [20, 25, 33–35], with 3 ml volume requiring between 35 and 67 s drying time [25, 33–35]. This could potentially cause a lack of compliance with recommended volumes and ineffective decontamination of hands. Indeed, Greenway et al. (2018) [41] report that regardless of the ABHR format, 3 ml volume was not perceived as acceptable by nurse participants, as it took too long to dry, could drip from the hands and resulted in the build-up of residue. Considering that frequently reported barriers to hand hygiene compliance in healthcare include lack of time, heavy workload and hand hygiene taking too much time [42–45], longer application times resulting from using larger ABHR volumes, which may be required for microbiological effectiveness, could lead to reduced acceptability and practicality of hand rubbing and subsequently, decreased hand hygiene compliance.

Application time is important though, not only for its relationship with the volume of ABHR used but also because it allows for contact between the active ingredient in the product used and contaminants on the hands. Evidence is also inconsistent with regards to the required ABHR application time, with only one study showing that 30 s was significantly more effective in reducing bacterial load on hands than 15 s [30], and another four studies demonstrating the lack of significant difference between the two application times [16, 18] or non-inferiority of the 15 to 30 s application time [23, 24]. Both clinically based studies that focused on the influence of ABHR application time on bacterial load reduction showed no significant difference between 15- and 30-s application time [16, 18]. It could be argued that unlike evidence derived from laboratory-based experiments, clinically based studies, conducted within natural, “real-life” conditions would have greater practical consequences. Furthermore, shortened application time could be practical and time-saving for HCWs; hence, improving compliance with hand hygiene guidelines. Nevertheless, the sample size in each of the two clinical studies was small (n = 14 each) [16, 18], which offers insufficient evidence to recommend shortening ABHR application time to 15 s. Thus, current WHO and CDC recommendations for ABHR application time of between 20 and 30 s [2] or of about 20 s [5] seems pragmatic in the light of the evidence published on this matter.

One possible solution to increase volume and application time in clinical practice is to standardise dispensers to ensure the required ABHR volume is delivered by a single dispenser action. A recent observational study of the ABHR volumes showed that the average ABHR volume used was only 1.09 ml (SD: 0.61), a dose similar to that delivered by the hospital’s automated ABHR dispensers (1.1ml) [46]. Yet, a recent study [47] investigating the dispensing performance of 22 wall-mounted ABHR dispensers commonly used in hospitals showed that the ABHR volume delivered by the dispensers was influenced by the ABHR format, the level of ABHR present in the container and the time lapse between dispenser uses. Furthermore, ABHR volume and contact time required to effectively reduce bacterial load on hands, as well as drying rate vary between the ABHR products, depending on their composition and alcohol concentration [3, 19, 30]. Thus, universal standardisation of ABHR dispensers might not be feasible. Furthermore, standardising ABHR volume delivered by the dispensers would not allow for adjusting the ABHR dose according to hand size. Although reviewed evidence on the relationship between hand size and ABHR volume showed inconsistent findings, with only one [29] out of four studies demonstrating a significant relationship, the evidence was limited by small sample sizes, which could result in a narrow range of hand sizes insufficient to detect a significant relationship between the variables. This requires further research and innovation in ABHR dispenser design to take account of this need.

Our systematic review provides an important and up to date contribution to the body of knowledge on factors influencing the effectiveness of hand rubbing with ABHR. It provides a unique evidence synthesis focusing specifically on hand rubbing with ABHR. It has used rigorous methods and all studies included in our review used study designs that met the EPOC criteria [13]. We have also reviewed and reported the review in adherence with the PRISMA statement [9] to enhance the rigour of our review.

Some challenges were experienced with the application of eligibility criteria during the study selection process. In our review, we included humans in the context of clinical practice or laboratory settings, but excluded studies conducted in healthcare settings in which participants were patients or visitors. This created a concern whether laboratory-based studies’ samples, described as volunteers could possibly include patients or visitors. However, because most of the laboratory-based studies used testing standards, such as EN1500 which defines participants as “healthy volunteers”, [48] we considered it unlikely for these samples to include patients. In addition, we assumed that for practical, ethical and accessibility reasons, laboratory-based studies’ samples were unlikely to include hospital patients or visitors. Furthermore, in our review, we included studies that focused on hand rubbing with ABHR, regardless of the format (i.e. liquid, gel or foam). Yet, in our search strategy, we have only included search terms related to ABHR in general and ABHR gels. However, we believed that the use of broad “ABHR” index terms and search terms would have covered for different ABHR formats.

Some limitations are acknowledged in respect to the search strategy. Language restrictions were applied, which could result in relevant studies being omitted. Furthermore, with the exception of one study, all studies included in the review were conducted in high-income countries; thus, studies from low and middle-income countries were underrepresented. While this underrepresentation could result from language restrictions applied to our search, another hand hygiene systematic review in which no language restrictions were applied, also identified most studies to be conducted in high-income countries [39, 40].

In addition, there are limitations in the evidence base. There was a substantial heterogeneity across the studies in application time, volume, application technique and ABHR product used. All studies were also assessed as being high risk of bias. The identified methodological limitations of the studies have been detailed and should be carefully considered and addressed in future research. Furthermore, most studies included in the review were conducted in laboratory settings. While such settings offer highly controlled conditions and allow for a greater level of intervention standardisation, they do not reflect real practice conditions, limiting generalisability.

Current guidelines recommend using a palmful of ABHR, sufficient to cover all hand surfaces within 20 to 30 s [2] or using a manufacturer-recommended volume for rubbing hands until dry for about 20 s while covering all surfaces [4, 5]. From this systematic review, there is a lack of high-quality evidence to make recommendations for changes. Thus, current guidelines should be followed until the body of evidence can be developed.

## Conclusions

Our systematic review demonstrates that complex relationships exist between ABHR volume, hand size, application time and bacterial reduction factor. For clinical practice, the situation is even more complex as these factors need to be considered alongside their practicality, acceptability and feasibility. Considering the substantial heterogeneity of the knowledge base, its limited methodological quality and some inconsistencies in the findings, the evidence is insufficient to advise any changes to current guidance. Future hand hygiene research in this field should consider ABHR volume, application time and hand size as potential confounding factors in study design considerations in order to build a more homogenous body of evidence that could inform international hand hygiene guidance.

## Supplementary Information

**Additional file 1**: Search strategy applied to MEDLINE database.

**Additional file 2**: Data extraction tool.

**Additional file 3**: Risk of bias of included studies.

## Data Availability

Extraction data and quality assessments that support the findings of this study are available from the corresponding author upon reasonable request.

## Abbreviations

ABHR: Alcohol-based handrub
CDC: Centers for Disease Control and Prevention
EPOC: Effective Practice and Organization of Care
GRADE: Grading of Recommendations Assessment, Development and Evaluation
HCWs: Healthcare workers
MRSA: Methicillin-resistant *Staphylococcus aureus*
NRTs: Non-randomized trials
RCTs: Randomized Controlled Trials
WHO: World Health Organisation
