# Supplementary material for: Systematic review on factors influencing the effectiveness of alcohol-based hand rubbing in healthcare
Source: Antimicrob Resist Infect Control. 2022 Jan 24;11:16. doi: 10.1186/s13756-021-01049-9 (PMC8785453; doi:10.1186/s13756-021-01049-9)
Supplement: Supplementary file 1 — Additional file 1: Search strategy applied to MEDLINE database. [file 13756_2021_1049_MOESM1_ESM.docx]

# Supplementary file I

***Search strategy applied to MEDLINE database***

| Search Terms | |
| --- | --- |
| 1 | (MH "Hand Sanitizers") |
| 2 | (MH “Hand Disinfection”) |
| 3 | AB, TI(ABHR OR "alcohol-based hand-rub*" OR "alcohol-based handrub*" OR "alcohol based hand rub*" OR "alcohol based handrub*" OR "alcohol rub*" OR "alcohol handrub*" OR "alcohol hand-rub*" OR "alcohol hand rub*" OR "alcohol-based hand gel*" OR "alcohol based hand gel*" OR "alcohol-based gel*" OR "alcohol based gel*" OR "alcohol gel*" OR "hand saniti?er*" OR saniti?er* OR "waterless antiseptic agent*" OR ABHS) |
| 4 | AB, TI ("hand disinfection" OR hand-rubbing OR handrubbing OR "hand rubbing" OR "hand hygiene" OR "hand asepsis" OR "hand antisepsis" OR "hand decontamination" OR "hand degerming" OR "hand sanitation") |
| 5 | AB, TI (Ayliffe* OR Ayliffe’s OR 6-step* OR "6 step*" OR six-step* OR "six step*" OR WHO OR WHO’s OR "world health organi?ation*" OR 7-step* OR "7 step*" OR 3-step* OR "3 step*" OR three-step* OR "three step*" OR CDC OR CDC’s OR "Centre for Disease Control and Prevention" OR "reasonable application") |
| 6 | AB, TI (technique* OR approach* OR method* OR practice* OR procedure* OR guideline* OR protocol* OR recommendation*) |
| 7 | AB, TI (time* OR duration* OR period OR length OR minute* OR second*) |
| 8 | AB, TI (friction* OR pressure* OR force*) |
| 9 | AB, TI (volume* OR amount* OR quantit* OR measure OR portion*) |
| 10 | AB, TI (“hand size” OR “surface area”) |
| 11 | (MH “colony count, microbial+”) OR (MH “bacterial load”) |
| 12 | AB, TI ("bacterial load" OR "antibacterial efficacy" OR "colony count" OR contamination OR colonization OR "colony forming units" OR cfu OR “microbial count” OR “microbial load” OR “microbial contamination” OR “microbial coloni?ation” OR “bacterial count” OR “bacterial contamination” OR “bacterial coloni?ation” OR “skin flora”) |
| 13 | AB, TI ("hand cover*" OR "surface cover*" OR "anatomical sites cover*" OR "hand cover*" OR "area* missed" OR "surface* missed" OR "anatomical site* missed" OR cover* OR UV OR ultraviolet) |
| 14 | AB, TI ("surgical scrub" OR "surgical scrubbing" OR "surgical hand disinfection" OR "surgical hand decontamination" OR "surgical hand hygiene" OR "surgical hand antisepsis" OR "surgical hand preparation" OR "perioperative scrubbing" OR "perioperative hand disinfection" OR "perioperative hand hygiene" OR "perioperative hand hygiene" OR "perioperative hand preparation" OR "preoperative scrubbing" OR "preoperative hand decontamination" OR "preoperative hand hygiene" OR "preoperative hand antisepsis" OR "preoperative hand preparation" OR "operating theater" OR "operating theatre" OR "operating department") |
| 15 | S1 OR S2 OR S3 OR S4 |
| 16 | S5 OR S6 OR S7 OR S8 OR S9 OR S10 |
| 17 | S7 OR S11 OR S12 OR S13 |
| 18 | (S15 AND S16 AND S17) NOT S14 |
| 19 | Limiters applied to S18 search:  Date of Publication: March 2019 – 2021; English Language |
