# Supplementary material for: Systematic review on factors influencing the effectiveness of alcohol-based hand rubbing in healthcare
Source: Antimicrob Resist Infect Control. 2022 Jan 24;11:16. doi: 10.1186/s13756-021-01049-9 (PMC8785453; doi:10.1186/s13756-021-01049-9)
Supplement: Supplementary file 2 — Additional file 2: Data extraction tool. [file 13756_2021_1049_MOESM2_ESM.docx]

# Supplementary file II

***Data extraction tool***

| **Study ID** | **Aim** | **Settings** | | **Design** | **Population** | | | |
| --- | --- | --- | --- | --- | --- | --- | --- | --- |
| Author, year | Aim(s) | Country | Settings | Design | Population of interest | Inclusion criteria | Exclusion criteria | Sample size |
|  |  |  |  |  |  |  |  |  |

| **Intervention/area of interest** | | | | **Standardisation/fidelity** | | | | |
| --- | --- | --- | --- | --- | --- | --- | --- | --- |
| Intervention/area of interest | Control/comparator (if relevant) | Sampling strategy | Product (type of alcohol, concentration, format - e.g., gel, foam, liquid) | Technique | Training | ABHR volume standardised? | Time standardised? | ABHR product standardised? |
|  |  |  |  |  |  |  |  |  |

| **Data collection** | | | | | | **Outcomes** |
| --- | --- | --- | --- | --- | --- | --- |
| Microbiological data collection methods (if relevant): were hands artificially contaminated? | Microbiological data collection methods (if relevant): sampling technique | Microbiological data collection methods (if relevant): were neutralisers used? | Other data collection methods | Data collection points (e.g., Before, during and after HH) | interrater variability reported? (If relevant) yes/no | Study outcomes (relevant to the review) |
|  |  |  |  |  |  |  |

| **Analysis** | | | **Results** | | | **Limitations** | **Conclusions** | **Other comments** |
| --- | --- | --- | --- | --- | --- | --- | --- | --- |
| Analysis | Power analysis? Yes/no | Hand size taken into account in the analysis? | Participants' demographic data | Study results | Compliance with HH protocol (if available) | Limitations | Authors' conclusions | Other comments |
|  |  |  |  |  |  |  |  |  |
