# Supplementary material for: Systematic review on factors influencing the effectiveness of alcohol-based hand rubbing in healthcare
Source: Antimicrob Resist Infect Control. 2022 Jan 24;11:16. doi: 10.1186/s13756-021-01049-9 (PMC8785453; doi:10.1186/s13756-021-01049-9)
Supplement: Supplementary file 3 — Additional file 3: Risk of bias of included studies. [file 13756_2021_1049_MOESM3_ESM.docx]

# Supplementary file III

***Risk of bias of included studies***

| Study ID | Adequate sequence generation? | Allocation concealment? | Baseline outcome measurements similar? | Baseline characteristics similar? | Incomplete outcome data addressed? | Blinding? | Protected against contamination? | Selective outcome reporting? | Free of other bias? | **Overall risk of bias** |
| --- | --- | --- | --- | --- | --- | --- | --- | --- | --- | --- |
| **Studies investigating ABHR volume** | | | | | | | | | | |
| Macdonald et al. (2006) | H | H | U | L | L | H | U | L | U | **H** |
| Kampf (2008) | U | H | L | L | U | U | H | L | U | **H** |
| Rotter et al. (2009) | U | H | U | L | L | U | H | L | U | **H** |
| Goroncy-Bermes, Koburger & Meyer (2010) | H | H | U | L | U | U | H | L | U | **H** |
| Girard et al. (2012) | H | H | U | U | U | H | L | L | U | **H** |
| Kampf et al. (2013) | U | H | U | L | L | L | H | L | U | **H** |
| Li et al. (2014) | U | U | L | H | U | U | H | H | U | **H** |
| Macinga et al. (2014) | U | H | U | L | U | U | H | H | U | **H** |
| Bellissimo-Rodrigues et al. (2016) | H | H | U | L | L | U | H | L | U | **H** |
| Wilkinson et al. (2017) | H | H | U | L | U | U | H | L | U | **H** |
| Jain, Clezy & McLaws (2018) | H | H | U | L | L | U | H | L | U | **H** |
| Suchomel et al. (2018) | H | H | U | L | L | U | H | L | U | **H** |
| Wilkinson et al. (2018) | H | H | U | L | U | L | H | L | U | **H** |
| Kenters et al. (2019) | H | H | U | L | U | U | H | H | U | **H** |
| **Studies investigating ABHR application time** | | | | | | | | | | |
| Dharan et al. (2003) | H | H | L | L | L | U | H | L | U | **H** |
| Rotter et al. (2009) | U | H | U | L | L | U | H | L | U | **H** |
| Kramer et al. (2017) | U | U | L | H | U | U | H | L | U | **H** |
| Pires et al. (2017) | U | H | U | L | H | U | H | L | U | **H** |
| Pires et al. (2019) | L | H | L | L | U | L | H | L | U | **H** |
| Harnoss et al. (2020) | L | H | L | L | L | H | U | L | U | **H** |
| **Study investigating rubbing friction** | | | | | | | | | | |
| Tan et al. (2020) | U | H | U | L | U | U | H | L | U | **H** |

ABHR=alcohol-based handrub; H=high risk; L=low risk; U=unclear risk
